# Non-homogeneity in the efficiency evaluation of health systems

**DOI:** 10.1186/s12913-023-10246-8

**Published:** 2023-11-10

**Authors:** Martin Dlouhý

**Affiliations:** Faculty of Statistics and Informatics, Prague University of Economics and Business, Prague, Czech Republic

**Keywords:** Health systems, Data envelopment analysis, Efficiency evaluation, Non-homogeneity

## Abstract

**Background:**

An international comparison of health system performance is a popular tool of health policy analysis. However, the efficiency evaluation of health systems is a practical example of an international comparison in which non-homogeneity is expected. The objective of this paper is to evaluate the efficiency of health systems by models in which a degree of non-homogeneity among countries is considered.

**Methods:**

We study the problem of non-homogeneity of health systems in the theoretical framework of the data envelopment analysis (DEA), which is a popular method of efficiency evaluation with hundreds of applications from various fields. DEA assume the homogeneity of production units and the homogeneity of the environment in which the production units operate. Hence, we compiled a summary of 14 recommendations on how to deal with the non-homogeneity in the DEA models. The analysed sample includes 38 OECD member countries. The data are from the year 2019.

**Results:**

As an example, we evaluated the health system efficiency of the Czech Republic. We used the DEA models with the neighbourhood measure of distance and the constraint limiting the comparison of countries with different levels of economic development. The health system inputs were the numbers of physicians, nurses, and hospital beds. In the production of the intermediate outputs (doctor consultations, inpatient care discharges), the Czech Republic should look at Poland, Slovakia and Slovenia. In the production of health outcomes (life expectancy), the peer countries are France, Italy and Switzerland.

**Conclusions:**

The results of the DEA analysis are only indicative because no single analytical method can determine whether a health system is better or worse than others. We need to combine different methods, and DEA is one of them. We consider DEA as an exploratory method, not a method providing definitive answers.

## Introduction

An international comparison of health system performance is a popular and frequently used tool of health policy analysis. Lessons from the top-performing countries can inform the decision-makers seeking to improve the national health system. International comparisons, including national health system performance assessments with international benchmarks, are carried out by the World Health Organization [1, 2], the European Observatory on Health Systems and Policies [3–5], OECD [6], the European Commission [7], as well as by the universities, research centres, and think tanks [8–14].

In our view, there are three assumptions of international comparisons. Firstly, health systems are comparable, so their production processes are more or less homogeneous. The issue of comparability (homogeneity) of health systems is the main focus of this paper. Secondly, we are able to say that the performance of one health system is, at least in some aspects, better than the performance of another health system. Otherwise, such performance would be useless. Thirdly, the international experience obtained from performance evaluation is, to some extent, transferable from one health system to another.

There are various ways to evaluate the performance of health systems. Still, none of them provides a universal guide to determining whether a health system is better or worse compared to other health systems. One of the key findings of the European Commission Report [7] is that no single efficiency metric at any level of analysis can generally indicate whether an evaluated entity is efficient. To get a more comprehensive analysis, it is appropriate to combine different methods. Data envelopment analysis (DEA), a popular quantitative method of relative efficiency evaluation, may be one of the complementary methods. Mbau et al. [15] reviewed 131 papers that evaluated the efficiency of health systems on a national or subnational level. DEA was used exclusively in 76% of the papers and used in 2% of the papers in combination with Free Disposal Hull and stochastic frontier analysis.

Efficiency evaluation in health care is a challenging application field with many specific characteristics. Above all, the causal relationship between health system inputs and health as the final output is uncertain. Many health determinants are outside the health system, such as lifestyle, physical activity, smoking, and alcohol consumption. Hence, the definition of the national health system as a traditional production unit in DEA is a simplification that allows us to study this system, but we must be aware of many limitations. Measuring the health system efficiency by using intermediate outputs such as the number of consultations and hospitalisations ignores health as the ultimate goal but allows us to think more about the health system as a production unit.

The technical efficiency is used in studying how health care can be produced when both outputs and inputs are measured in physical units. An allocation of resources is technically efficient if no alternative allocation would increase the output of one good without reducing the output of others. We can define the technical efficiency of a health system in two ways: first, efficiency is defined as the ratio between health system inputs (costs, in the form of labour, capital, or equipment) and outputs (e.g., the number of patients treated), and second, efficiency is defined as the ratio between health system inputs and health outcomes (e.g., life years gained). The health system is not a single production unit but an aggregation of many small units, such as medical practices, hospitals, nursing homes, pharmacies, etc. The units operate within a national health system that sets administrative and financial regulations. We measure the performance of this *system*.

The efficiency evaluation of health systems is a practical example of an international comparison in which a presence of non-homogeneity is expected. Hence, the comparability of health systems is a theoretical and practical problem. No health systems are perfectly homogeneous, so it is not practical to deal with non-homogeneity unless it exceeds a certain limit. Thus, one possibility is that a researcher ignores the problem and assumes for convenience that the health systems are homogeneous and comparable. Another possibility is that a researcher accepts the limited comparability of health systems and chooses an appropriate model or procedure to enhance the comparability.

In this paper, we study the problem of non-homogeneity of health systems in the theoretical framework of the data envelopment analysis. DEA is a popular method of efficiency evaluation with hundreds of applications from various fields, such as health care, education, banking, agriculture and others [16]. DEA assume the homogeneity of production units and the homogeneity of the environment in which the production units operate. However, such assumptions do not often correspond to reality. We investigate how the comparability of production units (health systems) can be enhanced in the case of non-homogeneity. The objective of this paper is to evaluate the efficiency of health systems by the DEA models in which a degree of non-homogeneity among countries is considered.

The rest of the paper is organised as follows: Non-Homogeneity in Data Envelopment Analysis section introduces DEA and makes a summary of recommendations on how to deal with non-homogeneity in DEA. An application section is an application to health system efficiency evaluation. Conclusion section concludes the paper.

## Non-homogeneity in data envelopment analysis

There are two types of main quantitative methods of efficiency evaluation. The first method is non-parametric data envelopment analysis [17], and the second method is the stochastic frontier analysis [18]. In this paper, we use the data envelopment analysis as it does need any assumption about the functional form of the production frontier, it comfortably deals with multiple inputs and outputs, and it is able to identify (for inefficient units) the peers that are real production units. DEA uses linear programming to construct the production frontier as the piecewise linear envelopment of the data. The method assumes that the homogenous production units use a set of inputs to produce a set of outputs and that the weights (prices) of inputs and outputs are unknown. Without information on prices, the allocative efficiency that measures the ability to use inputs in optimal proportions and the overall economic efficiency that is the product of technical and allocative efficiency cannot be calculated. DEA calculates the technical efficiency of a production unit as the best possible ratio of the weighted output to the weighted input or vice versa.

DEA classifies the production units as technically efficient or technically inefficient. Technically efficient units are those that lie on the production frontier and can serve as peer units for inefficient units. DEA distinguishes the output-oriented model, which maximises quantities of outputs produced by the fixed levels of inputs, and the input-oriented model, which minimises quantities of inputs required to produce the fixed levels of outputs. According to the character of the returns to scale, there are two original DEA models: the CCR model with the constant returns to scale [17], and the BCC model with the variable returns to scale [19]. Many other models have been developed since the formulation of the first DEA models [20–22].

The DEA models have two equivalent formulations: the multiplier form and the envelopment form. Suppose we have a set of *n* production units that use *m* types of inputs to produce *r* types of outputs. The envelopment formulation of the input-oriented variable-returns-to-scale DEA model (1) for production unit *q* is below:

1 $$\begin{array}{l}\begin{array}{l}\begin{array}{l}{minimise\;\theta}_q\\subject\;to\\\sum\nolimits_{j=1}^nx_{ij}\lambda_j\leq\theta_qx_{iq},\;i=1,2,...,m,\end{array}\\\sum\nolimits_{j=1}^ny_{kj}\lambda_j\geq y_{kq},\;k=1,2,...,r,\end{array}\\\lambda_j\geq0,\;j=1,\;2,...,n,\\\sum\nolimits_{j=1}^n\lambda_j=1,\end{array}$$

where *θ*_*q*_ is the technical efficiency score of unit *q*, *x*_*ij*_ is the quantity of input *i* used by unit *j*, *y*_*kj*_ is the quantity of output *k* produced by unit *j*, *λ*_*j*_ is the variable that measures the individual contribution of unit *j* in the formation of the efficient target for unit *q*. If ${\lambda }_{j}>0$, production unit *j* serves as an efficient peer unit for unit *q*. In the input-oriented DEA model, the technical efficiency score *θ*_*q*_ represents a size of input reduction that makes unit *q* technically efficient.

In the DEA framework, Dyson et al. [23] stated three assumptions of homogeneity: (1) the production units perform similar activities and produce comparable outputs; (2) the same set of inputs is available to all units; (3) the units operate in similar external environments. The first two assumptions of homogeneity are related to production units, while the third assumption is related to a non-homogeneous environment under which production units operate. The environment is defined as a set of external factors that affect the technical efficiency of a production unit but are not usually considered typical inputs in the DEA models and are not under the control of the management. Examples of such external factors are governmental regulation, socio-economic conditions, ownership, and geographic location.

We compiled a summary of 14 recommendations on how to deal with the non-homogeneity in the DEA models. Recommendations 1–7 deal with the non-homogeneity in the set of production units, and recommendations 8–14 deal with the non-homogeneity of the external environment.

**Fig. 1:**
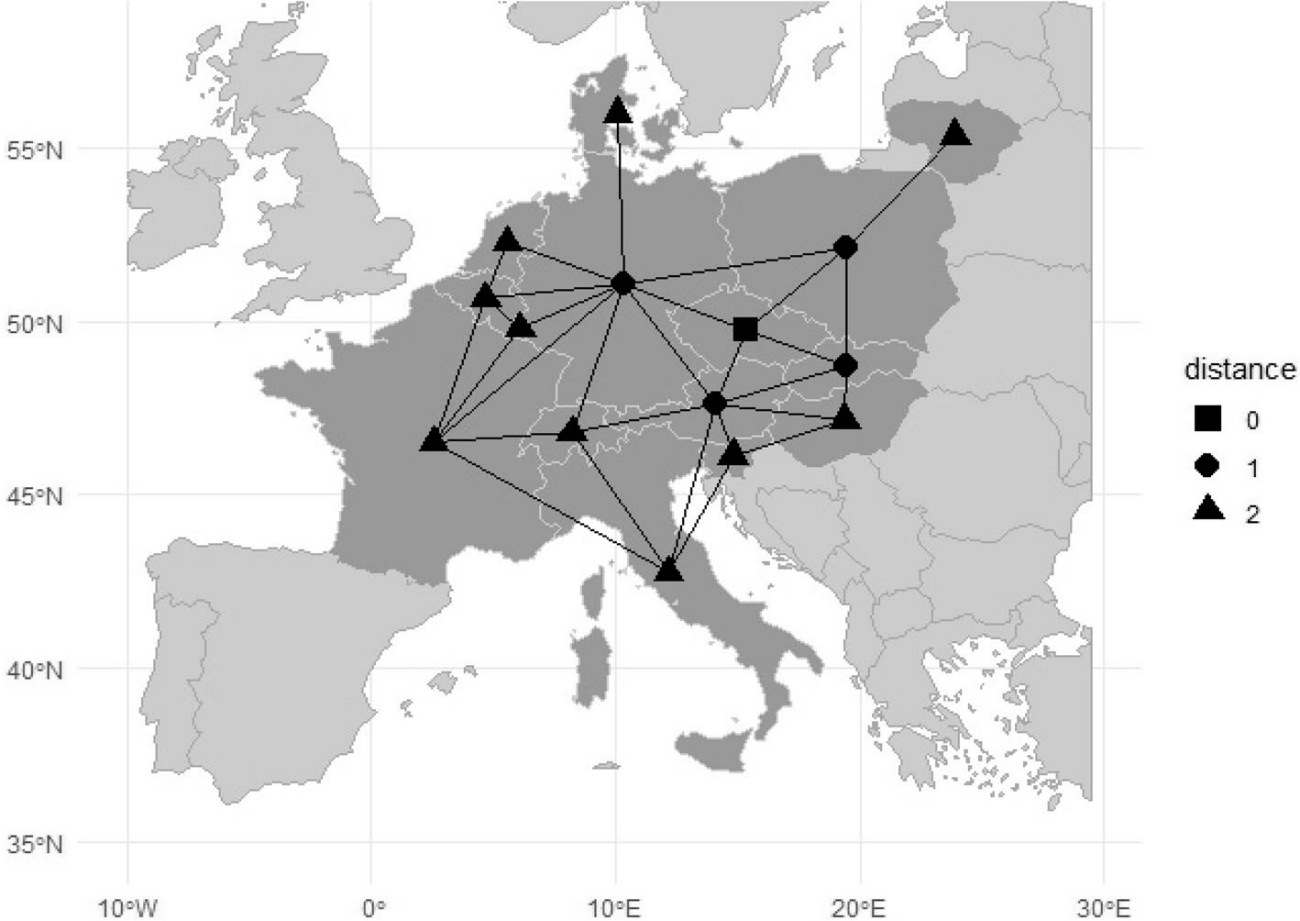
Neighbours of the Czech Republic within a maximum distance of 2

1. *Choose the set of units carefully*. A researcher can avoid the problem of non-homogeneity if the set of units is properly chosen. For example, different faculties from the same university can be compared internally, but they are not homogeneous in terms of inputs and outputs. Therefore, the correct procedure would be an external comparison with equally focused faculties from other universities [23].
2. *Divide the set of units into homogeneous categories*. Dividing the set of units is a frequent way to deal with non-homogeneity, especially if a sufficient number of units is at disposal. In this method, the categories must be specified by a researcher. In some cases, the criterion is relatively straightforward; for example, in hospital efficiency evaluation, it is reasonable to divide the set of hospitals into the category of teaching hospitals and the category of non-teaching hospitals [23]. In other cases, the categories are not explicitly given and have to be determined.
3. *Be aware of the limited validity of the evaluation*. Even if there are serious doubts about the homogeneity of production units, it is still possible to perform a DEA evaluation. However, the validity of the results should be subjected to a critical analysis [23].
4. *Select the right type of returns to scale*. Another source of non-homogeneity is a false assumption about the returns of scale. For example, the wrong choice of a model with the variable returns to scale instead of the one with constant returns to scale leads to an overestimation of efficiency scores for the smallest and largest units [23].
5. *Construct a unit-specific reference set*. Golany and Thore [24] describe the concept of dynamic clustering that establishes a categorisation of the set of units for each evaluated unit *q*. The set of units is divided into the subset *P*_*q*_ (permitted units) and the subset *N*_*q*_ (not-permitted units) for each unit *q*. Hence, each unit constructs its unit-specific frontier. The boundaries of the cluster can be defined in absolute terms, so the unit belongs to the cluster only if its distances from the evaluated unit are smaller than the predefined distance in all dimensions. The alternative possibility is to define the boundaries of the cluster in relative terms determined by the proportions taken from input–output values of the evaluated unit *q*. The following constraint on the cluster (unit-specific reference set) is added to the DEA model (1):
  2 $$\sum_{j\in {N}_{q}}{\lambda }_{j}=0.$$
6. *Use reliable data*. The ideal situation is if the data come from standardised databases and accounting systems. The non-homogeneity in the data can be a particular problem in studies involving international comparisons in which there is a risk of different national definitions of indicators. The exact definitions of data are given in a reliable database, and different definitions used in some countries are mentioned. We recommend using the databases of international organisations and statistical offices, such as the World Bank, OECD, Eurostat, and the World Health Organisation. These are the best sources of comparative data available.
7. *Use internal measures of distance.* We assume that the degree of homogeneity is a decreasing function of the distance between units. In this alternative, the distance between two production units (health systems) is based on all input and output values or a selected subset of them. We can follow Golany and Thore [24] and measure the distance separately for each input or output dimension, or we can construct a single (weighted) measure of distance. The simple case is if the data are normalised to the [0,1] range, and the distance between units is measured by the non-weighted Euclidean distance. We propose the following approach:
  1. *Determine the input and output weights*. The unit-specific weights of inputs and outputs are determined by the multiplier DEA model; alternatively, the weights are determined by the common weights model in which the weights of inputs and outputs are the same for all units. Both alternatives for determining weights are in accordance with the DEA methodology and should be prioritised over unweighted measures of distance.
  2. *Calculate the matrix of distances*. The weighted internal measures of distance *d*_*ij*_ are calculated for each pair of units. You do not have to include all inputs and outputs in your calculations. In the health system efficiency evaluation (Application section), the inputs are physicians, nurses, and hospital beds, and the outputs are doctor consultations and inpatient care discharges. If you want to compare the health systems with the same structure of health system inputs, you do not have to include outputs to calculate the distances between units. The distances are normalised by setting the maximum distance to one so that distances lie in the interval [0,1].
  3. *Apply the DEA model*. For each unit *q*, the envelopment BCC model is calculated with comparability constraints on the intensity variables ${\lambda }_{j}\le {d}_{qj}$.
    The comparability constraints on the intensity variables ${\lambda }_{j}$ can have various forms. Some examples for the variable returns-to-scale DEA model (1) and the normalised values *d*_*qj*_ follow:
    3a $$\lambda_j\leq1-{\mathrm\alpha d}_{qj}\;\text{for }j=1,2,\dots,n;0\leq\propto\leq1;$$
    3b $$\lambda_j\leq1-{{(d}_{qj})}^\beta\;\text{for }j=1,2,\dots,n;\beta>\;0;$$
    3c $$\lambda_j\leq e^{-\gamma d_{qj}}\;\text{for}\;j=1,2,\dots,n;\gamma\geq0;$$
    3d $$\lambda_j\leq ceil(\delta\left(1-d_{qj}\right))/\delta\;\text{for}\;j=1,2,\dots,n;\delta\in\mathbb{N}^+;$$
    where *d*_*qj*_ is the distance between the evaluated unit *q* and unit *j*; parameters *α*, *β*, *γ*, and $\delta$ are determined subjectively by the researcher, which makes it possible to flexibly determine the decreasing influence of units according to their distance from the unit *q*. In the CCR model, we modify (3a-3d) to allow values greater than 1. The disadvantage of this approach in some applications is that relatively distant units can also be selected as peer units. In situations where such a finding is undesirable, it is appropriate to combine comparability constraints (3a-3c) with constraint (2) to set the maximum allowable distance between the evaluated unit and peer units.
8. *If the environmental factors can be ordered, compare the production unit only with those units that are from the same category or the categories with a less favourable environment*. This approach prohibits situations in which a production unit is unfairly compared with units operating in a more favourable environment [25].
9. *In the case of the categorical character of the environmental factor, compare the production unit only with units from the same category*. In this case, a separate production frontier is calculated for each category of units. If we compare efficiency scores obtained from separate production frontiers with efficiency scores of the common production frontier, we can determine the effect of the external environment. Typical examples of external factors are public/private ownership and geographical location [25].
10. *If the environmental factors are continuous, include them directly in the model*. If an external factor is favourable, it should enter the DEA model as an input, assuming that more output is produced. If an external factor is unfavourable, it should enter the DEA model as an output, assuming that more input is needed. Because radial reduction of inputs or expansion of outputs of external factors does not make sense, it is recommended for the external factors to enter the model as the non-controllable inputs or outputs [25].
11. *If the environmental factors have a categorical or continuous character, use regression to identify environmental factors and measure their significance*. In the first stage, the DEA model with conventional inputs and outputs is calculated. In the second stage, the technical efficiency scores are regressed upon the environmental factors. The advantages are that the method can test if the environmental factor has a statistically significant influence on efficiency, the method can be used for more factors, and it does not need any prior assumptions on the direction of the influence of the environmental factor [25].
12. *Set the maximal proportions of units from other categories in the reference set*. Golany and Thore [24] studied the possibility of the restricted reference set in cases in which an analyst has to consider the external non-homogeneous environment (institutional circumstances, externalities in production, equity considerations or other extraneous information). The so-called *categorisation constraints* control the participation of units in the reference set for other units simply by separating the whole sample into clusters (groups). In the dichotomous approach, described by recommendation (9), we will either include or not include a unit in the subset of permitted units *P*_*q*_*.* However, a more flexible approach is proposed in which units from the set of non-permitted units *N*_*q*_ are partially included in the reference set by adding a constraint such as:
  4 $$\sum_{j\in N_q}\lambda_j/\sum\limits_{j=1}^n\lambda_j\leq\lambda_{max}^{N_q},$$
  where ${\lambda }_{max}^{{N}_{q}}$ is a constant that determines the maximal proportion of units from *N*_*q*_ in the reference set. If the maximal proportion constant ${\lambda }_{max}^{{N}_{q}}$ is set to zero, then units outside the category cannot be included in the reference set, and an evaluation of units according to individual categories is carried out.
13. *Use external measures of distance.* In this alternative, the distance is not derived from input or output values as in the case of the internal measures of distance (recommendation 7) but from some external variable that is independent of the production process and does not enter production directly. An external variable can be a geographical distance among cities or the gross domestic product in the case of an international comparison of health systems. The model is the same as for internal measures of distance, i.e. model (1) with constraints (3a-3d) proposed by recommendation (7). The difference is just in the type of non-homogeneity. We can easily combine internal and external measures according to the nature of the analysed problem.
14. *Use the neighbourhood measure.* The use of neighbourhood as a measure of distance is suitable for evaluating geographical units such as districts, regions, and countries. The distance *d*_*qj*_ between the evaluated unit *q* and unit *j* is measured in a graph in which vertices are the units and edges express the geographical neighbourhood (see Fig. 1 in Application section). The distance *d*_*qj*_ is the path between the units *q* and *j* with the minimal number of edges. While the geographical distance takes on continuous values, the neighbourhood distance takes on discrete values. The neighbourhood distance is 1 if the two geographical units are direct neighbours. The comparability constraint for unit *q* can be formulated in various ways; the following one (5a) can be the first choice for the variable returns-to-scale model:
  5a $$\lambda_j\leq\frac1{d_{qj}}\;\text{for}\;j=1,2,\dots,n,\;j\neq q.$$

This comparability constraint (5a) may be supplemented by a constraint (5b), which eliminates the influence of distant neighbours:

5b $$\lambda_j=0\;\mathrm{if}\;d_{qj}>D\;\text{for}\;j=1,2,\dots,\;n.$$

The first extreme case is when only direct neighbours enter the reference set (*D* = 1), then ${\lambda }_{j}$ can be positive for direct neighbours, and is zero for other units. The second extreme case is if *D* is greater than the maximal distance or equals infinity. In this case, all units are included in the evaluation.

## An application

Suppose that the Czech Ministry of Health asked a group of researchers to evaluate the performance of the Czech health system in comparison with *similar* health systems. One way for improvement is to compare the Czech health system with the health systems of countries with similar economic, social and cultural characteristics. The author chose the Czech Republic as an example because he works there.

Setting the health system goals is a crucial decision for any efficiency evaluation. Although there is some consensus on the most common goals of the health system, the efficiency evaluations differ in using intermediate goals (called as outputs) or final (health) system goals (called as outcomes). In the first model, *the output efficiency model*, the health system outputs are doctor consultations and inpatient care discharges. In the second model, *the outcome efficiency model*, the single health outcome is the life expectancy at birth. The data are from the year 2019 or the latest available year. The year 2019 was selected because the COVID-19 pandemic distorted the data from the following years. In both models, the health system inputs are the numbers of physicians, nurses, and hospital beds. The selected health system inputs and outputs are among the typical and most frequently used indicators [14]. However, we are aware of the problem that health is affected by various factors (health determinants) outside the health system. This will be partially addressed by including GDP in the model, but not as an input variable.

We reduced the non-homogeneity and thus enhanced the reliability of international comparison among health systems by the following recommendations. First, we choose the OECD member countries to obtain a set of developed countries with developed health systems (recommendation 1). Second, we use a reliable data source (recommendation 6), in this case, the *OECD Health Statistics*. The sample includes 38 OECD member countries (Table 1). Third, we apply the neighbourhood distance (recommendation 14), which is limited to a maximum distance of 2. Fourth, the possibility for countries to become peer units is limited by an external factor (recommendation 13), in this case by the gross domestic product (GDP) per capita in USD purchasing power parity. GDP expresses the power of the economy; however, GDP as an indicator of social and economic development also affects the quality of the health system.

**Table 1:** Health system data, the year 2019 or the latest available

Country	Physicians per 1000	Nurses per 1000	Beds per 1000	Consultations per person	Discharges per 1000	Life Expectancy	Neighbourhood measure	GDP per capita in USD	Normalised GDP distance
Australia	3.83	12.22	3.84	7.30	184	83.0	5	53,079	1
Austria	5.32	10.37	7.19	6.60	243	82.0	1	58,650	0.8
Belgium	3.16	11.07	5.57	7.30	165	82.1	2	54,710	1
Canada	2.74	9.98	2.52	6.60	82	82.1	5	50,666	1
Chile	2.64	2.87	2.03	2.90	86	80.6	5	25,764	0.8
Colombia	2.25	1.39	1.74	2.60	33	76.7	5	15,610	0.8
Costa Rica	3.07	3.41	1.10	2.30	53	80.5	5	21,748	0.8
Czech Republic	4.07	8.56	6.58	8.20	191	79.3	0	43,006	1
Denmark	4.19	10.10	2.59	4.00	146	81.5	2	60,335	0.8
Estonia	3.47	6.24	4.53	5.50	161	78.8	4	38,819	1
Finland	3.21	14.26	3.35	4.40	158	82.1	4	51,557	1
France	3.17	11.07	5.84	5.90	184	82.9	2	49,377	1
Germany	4.39	13.95	7.91	9.80	252	81.4	1	55,891	1
Greece	6.16	3.38	4.18	3.20	137	81.7	4	30,869	1
Hungary	3.49	6.62	6.91	10.70	190	76.4	1	33,957	1
Iceland	3.89	15.36	2.80	5.90	109	83.2	5	60,082	0.8
Ireland	3.32	12.88	2.88	5.80	134	82.8	4	89,431	0.6
Israel	3.29	5.01	2.96	8.20	150	82.9	5	41,948	1
Italy	4.05	6.16	3.16	10.40	113	83.6	2	44,851	1
Japan	2.49	11.76	12.84	12.50	131	84.4	5	42,230	1
Korea	2.46	7.94	12.44	17.20	180	83.3	5	42,728	1
Latvia	3.27	4.39	5.42	6.10	186	75.5	3	32,003	1
Lithuania	4.57	7.74	6.35	9.50	220	76.4	2	38,765	1
Luxembourg	2.98	11.72	4.26	5.50	143	82.7	2	120,962	0
Mexico	2.44	2.85	0.97	2.30	39	75.1	5	20,609	0.8
Netherlands	3.72	10.69	3.08	8.80	90	82.2	2	59,469	0.8
New Zealand	3.38	10.24	2.54	3.80	137	82.1	5	44,917	1
Norway	4.97	17.88	3.47	4.40	158	83.0	4	68,345	0.8
Poland	2.38	5.10	6.17	7.70	168	78.0	1	34,152	1
Portugal	5.32	7.08	3.51	4.10	107	81.8	4	36,872	1
Slovakia	3.57	5.74	5.76	11.10	189	77.8	1	32,557	1
Slovenia	3.26	10.28	4.43	6.70	173	81.6	2	41,194	1
Spain	4.40	5.89	2.95	7.30	114	83.9	3	42,184	1
Sweden	4.32	10.85	2.07	2.60	138	83.2	3	55,069	1
Switzerland	4.35	17.96	4.59	4.30	169	84.0	2	73,115	0.8
Turkey	1.95	2.40	2.88	9.80	165	78.6	4	27,600	1
United Kingdom	2.95	8.20	2.45	5.00	127	81.4	3	48,542	1
United States	2.64	11.98	2.83	4.00	125	78.9	5	65,298	0.8

In the first step, we solve *the output efficiency model* with the output orientation (6). As was already mentioned, the health system is not a typical production unit but an aggregation of many small units (e.g., hospitals, medical practices); thus, we decided to apply constant returns to scale. Our argument is based on the fact that the health system is not one firm that can increase its production with some type of returns of scale. The increased production is achieved by opening a new independent medical practice or a hospital. In *the output efficiency model*, three countries are technically efficient: Korea, Sweden, and Turkey (Table 2). The health system efficiency of the Czech Republic is 55%, with Turkey as a single peer. This result means that the Czech health system could reduce the amount of health system inputs by 45%, or alternatively, it should almost double outputs (doctor consultations and inpatient care discharges). This is not a very realistic health policy recommendation. The efficiency of 55% is extremely low, and the Ministry of Health will surely consider it as an inadequate benchmark. The inputs of the Turkish health system are relatively very low in comparison to other OECD member countries, and the outputs are quite high (Table 1). The reason may be differences in national reporting of health system outputs. The DEA study [13] also identified Turkey as an outlier compared to other OECD member countries. However, for comparison, we also calculated the output efficiency model with the variable returns to scale. This model is not very discriminatory because 13 health systems were identified as efficient (Table 2). The health system efficiency of the Czech Republic is 87%, with Austria, Germany, Lithuania, and Turkey as peers.

**Table 2:** Efficiency of health systems

Country	Output efficiency model with CRS (CCR model)	Output efficiency model with VRS (BCC model)	Outcome efficiency model with NIRS (life expectancy)	Outcome efficiency model with NIRS (HAQ Index)
Australia	0.8054	1.0000	0.9930	0.9815
Austria	0.5845	1.0000	0.9748	0.9638
Belgium	0.6141	0.7891	0.9848	0.9491
Canada	0.7697	0.7870	1.0000	1.0000
Chile	0.7195	0.7661	1.0000	1.0000
Colombia	0.4581	1.0000	1.0000	1.0000
Costa Rica	0.7988	1.0000	1.0000	1.0000
Czech Republic	0.5543	0.8700	0.9431	0.8996
Denmark	0.8876	0.9396	0.9766	0.9413
Estonia	0.6115	0.8230	0.9449	0.8792
Finland	0.7972	0.9068	0.9904	0.9566
France	0.6853	0.8819	0.9935	0.9644
Germany	0.6781	1.0000	0.9672	0.9396
Greece	0.5890	0.7818	1.0000	1.0000
Hungary	0.6435	0.9563	0.9145	0.8452
Iceland	0.6657	0.6697	0.9984	1.0000
Ireland	0.7699	0.8117	0.9991	0.9803
Israel	0.8708	0.9013	1.0000	1.0000
Italy	0.9672	1.0000	0.9996	1.0000
Japan	0.7182	0.7269	1.0000	1.0000
Korea	1.0000	1.0000	1.0000	1.0000
Latvia	0.6702	1.0000	0.9178	0.8680
Lithuania	0.6011	1.0000	0.9089	0.7514
Luxembourg	0.5829	0.7519	0.9983	0.9594
Mexico	0.7002	1.0000	1.0000	0.9882
Netherlands	0.8397	0.8603	0.9866	0.9980
New Zealand	0.8764	0.8926	0.9929	0.9414
Norway	0.7300	0.8933	0.9889	0.9710
Poland	0.8335	0.9309	0.9610	0.9579
Portugal	0.5079	0.6022	0.9746	0.9309
Slovakia	0.6247	1.0000	0.9338	0.8510
Slovenia	0.6756	0.8944	0.9807	0.9650
Spain	0.7272	0.7353	1.0000	1.0000
Sweden	1.0000	1.0000	1.0000	1.0000
Switzerland	0.6212	0.8555	1.0000	0.9946
Turkey	1.0000	1.0000	1.0000	1.0000
United Kingdom	0.8531	0.8614	0.9910	0.9601
United States	0.7503	0.7666	0.9652	0.9203

In *the outcome efficiency model* with the output orientation (7), the non-increasing returns to scale were applied, assuming that the health benefits of health system inputs are decreasing. In this model, the health systems of 13 countries are efficient (Table 2). The health system efficiency of the Czech Republic is 94%, with Japan, Spain, and Switzerland as peer countries. In this case, the health system efficiency of 94% looks reasonable. However, the Ministry of Health can consider it inadequate to have as a benchmark geographically and culturally distant country such as Japan. For a comparison, we also calculated the outcome efficiency model with the Healthcare Access and Quality Index (HAQ) as the output variable. The HAQ Index is measured on a scale from 0 (worst) to 100 (best) and uses 32 causes of amenable mortality that could be avoided by timely and effective health care [26]. In this model, 13 health systems were identified as efficient (Table 2). With the exception of Lithuania, the model with the life expectancy and the model with the HAQ index give similar results. The health system efficiency of the Czech Republic is 90%, with Canada, Iceland, and Spain as peers.

6 $$\begin{array}{l}{maximise\;\theta}_q\\subject\;to\\\begin{array}{l}\sum\nolimits_{j=1}^{38}x_{ij}\lambda_j\leq x_{iq},\;i=1,2,3,\\\sum\nolimits_{j=1}^{38}y_{kj}\lambda_j\geq{\theta_qy}_{kq},\;k=1,2,\\\lambda_j\geq0,\;j=1,2,...,38.\end{array}\end{array}$$

7 $$\begin{array}{l}{maximise\;\theta}_q\\subject\;to\\\begin{array}{l}\sum\nolimits_{j=1}^{38}x_{ij}\lambda_j\leq x_{iq},\;i=1,2,3,\\\sum\nolimits_{j=1}^{38}y_{kj}\lambda_j\geq{\theta_qy}_{kq},\;k=1,\\\begin{array}{l}\lambda_j\geq0,\;j=1,2,...,38,\\\sum\nolimits_{j=1}^{38}\lambda_j\leq1.\end{array}\end{array}\end{array}$$

Based on the results of the models, we decided to reduce the number of countries to ensure a comparison of the Czech health system with the more homogeneous set of health systems. In the second step, we introduce the neighbourhood distance measure (5b) that eliminates the influence of geographically distant countries. This neighbourhood distance is 1 if the two countries are direct neighbours. For overseas countries, we set the distance to 5 (Table 1). In the model, we set the maximum neighbourhood distance to 2 as a suitable limit on the proximity of two countries. The Czech Republic has 18 neighbours within a maximum distance of 2 (*d*_*qj*_ ≤ 2), see Fig. 1. Four countries are not the OECD member countries (Belorussia, Lichtenstein, Russia, and Ukraine) and are not included in the sample. In *the output efficiency model*, six health systems are efficient. The health system efficiency of the Czech Republic is 86%, with peer countries Poland, Slovakia, and Slovenia. The value of health system efficiency and the set of peer countries are more reasonable in comparison with the previous output efficiency model. In *the outcome efficiency model*, seven health systems are efficient. The efficiency score of the Czech Republic is 95%, with peer countries France, Italy, and Switzerland. The peer countries are now more suitable for the Czech Republic, and target values are much more achievable.

In the third step, we calculated the DEA models (8) and (9) that also consider normalised differences in the level of GDP (external factor), which is an important indicator of social and economic development. The constraint (4d) with *δ* = 5 was introduced into both models (see Table 1). These additional comparability constraints do not affect the efficiency of the Czech health system (86% and 95%), and the peer countries are also the same as in the previous output and outcome efficiency models with the neighbourhood distance measure. In Table 1, we can observe the imperfection of the constraint (3d), which assigns zero to the most distant unit (Luxembourg) and excludes that unit from the set. This can be corrected by the condition that the most distant unit gets the value of the last category 1/*δ*, which is 0.2 in this case.

Our health policy recommendation is that in the case of output production (consultations, inpatient care discharges), the Ministry of Health should look at Poland, Slovakia and Slovenia, and in the case of health outcomes (life expectancy) at France, Italy and Switzerland. However, this recommendation is only partial and indicative. As mentioned above, no single method provides a universal guide to determining whether a health system is better or worse compared to other health systems. It is appropriate to combine different methods, and DEA is one of them. We consider DEA as an exploratory method, not a method providing definitive answers. In our opinion, the DEA models used in this analysis that aim to reduce non-homogeneity among the health systems are superior to conventional DEA models due to more reasonable values of estimated health system efficiency and more reasonable selection of peer countries. This will improve the acceptance of the results by end users.

8 $$\begin{array}{l}\begin{array}{c}\begin{array}{l}{maximise\;\theta}_q\\subject\;to\\\sum\nolimits_{j=1}^{38}x_{ij}\lambda_j\leq x_{iq},\;i=1,2,3,\end{array}\\\sum\nolimits_{j=1}^{38}y_{kj}\lambda_j\geq{\theta_qy}_{kq},\;k=1,2,\\\lambda_j\geq0,j=1,2,...,38,\end{array}\\\lambda_j=0\;\mathrm{if}\;d_{qj}>2j=1,2,...,38,\\\begin{array}{l}\lambda_j\leq ceil(5\left(1-{normalised\;GDP\;distance}_{qj}\right))/5j=1,2,...,38,\\{normalised\;GDP\;distance}_{qj}=\frac{{abs({GDP}_q-GDP}_j)}{{\mathit{max}}_j{abs({GDP}_q-GDP}_j)}.\end{array}\end{array}$$

9 $$\begin{array}{c}{maximise\;\theta}_q\\subject\;to\\\begin{array}{c}\sum\nolimits_{j=1}^{38}x_{ij}\lambda_j\leq x_{iq},\;i=1,2,3,\\\sum\nolimits_{j=1}^{38}y_{kj}\lambda_j\geq{\theta_qy}_{kq},\;k=1,\\\begin{array}{c}\lambda_j\geq0,\;j=1,2,...,38,\\\sum\nolimits_{j=1}^{38}\lambda_j\leq1,\\\begin{array}{c}\lambda_j=0\;\mathrm{if}\;d_{qj}>2j=1,2,...,38,\\\begin{array}{c}\lambda_j\leq ceil(5\left(1-{normalised\;GDP\;distance}_{qj}\right))/5j=1,2,...,38,\\{normalised\;GDP\;distance}_{qj}=\frac{{abs({GDP}_q-GDP}_j)}{{\mathit{max}}_j{abs({GDP}_q-GDP}_j)}.\end{array}\end{array}\end{array}\end{array}\end{array}$$

## Conclusion

International comparisons of health systems allow us to study different approaches to delivering health care. However, at the international level, the comparability and efficiency evaluation of health systems is a theoretical and practical problem. As it was aptly said [12]: no two countries are alike when it comes to health care. Over time, each country has settled on a unique mix of policies, service delivery systems, and financing models.

In this study, we used data envelopment analysis, which is a popular quantitative method of efficiency evaluation in health care [15, 16, 27]. There are many other studies that evaluate the health system efficiency of the OECD member countries by DEA. For example, Retzlaff-Roberts et al. [28] included in their study 27 OECD member countries and used the OECD data for 1998. They computed both the input-oriented and output-oriented BCC models with seven inputs (four health system inputs and four social environment inputs) and one health system output. As the output they used infant mortality and life expectancy, so finally, they computed four different DEA models. Cetin and Bahce [13] evaluated the health system efficiency of 34 OECD member countries by the input-oriented CCR and BCC models. The number of doctors, number of beds, and health expenditure per capita were used as health system inputs and life expectancy at birth and infant mortality rate were used as outputs (year 2011). At the second stage of their analysis, eight countries were removed from the set as outliers. Cylus et al. [29] proposed the use of DEA to construct composite health system efficiency indicators from several partial efficiency measures. They tested their approach on a sample of 11 OECD member countries. Gavurova et al. [30] used the input-oriented dynamic network DEA model to compare the health system efficiency of 36 OECD member countries in the years 2000, 2008, and 2016. In their study, the health system was divided into the public health sub-division and the medical care sub-division. Pereira et al. [31] applied a quite complex network DEA model to evaluate the health system efficiency in the fight against COVID-19. The sample included 55 countries (37 OECD member countries, six prospective OECD members, four OECD key partners, and eight other countries). Behr and Theune [32] investigated the health system efficiency of 34 OECD member countries. Instead of analysing the whole health system, they conducted five separate partial analyses. They applied the input-oriented DEA with the constant returns to scale.

In this study, we focused on the problem of non-homogeneity of health systems in the theoretical framework of the data envelopment analysis. All the studies mentioned above [13, 28–32] included the whole set of available OECD countries in the health system efficiency evaluation by the DEA models without any restrictions. In contrast, in our study, comparisons of countries are limited by the neighbourhood distance and the level of economic development (GDP), which serve as a proxy measures of non-homogeneity. This is one of our contributions to the existing literature on the health system efficiency evaluation. The key difference between the DEA models used in this paper and the dynamic clustering [24] is that the inclusion of units in the reference set is not dichotomous but gradually decreases with the degree of expected non-homogeneity. To the best of our knowledge, the idea of the neighbourhood distance measure was not previously used in the DEA framework for the health system evaluation.

In addition to the theoretical contribution, the paper contains an application in which we look for suitable foreign health systems as models for the Czech health system. Extended DEA models in this study restrict the reference set in such a way that peer countries are more reasonable for the Czech Republic, and target values are better achievable. Hence, we believe that the recommendations proposed in this paper that aim to reduce non-homogeneity are superior to conventional DEA models mainly due to more reasonable results and their better acceptance by users. The author’s own personal experience as a health policy consultant supports this point of view that an unrealistic benchmark is not very inspiring and may be completely unsuitable for the practice.

There are some limitations of this study. First, there needs to be a higher number of health system input and output indicators in the DEA models, which is limited by the number of units in the sample. Second, the limited availability of international data and its comparability are traditional problems of international comparisons. Third, outliers can extremely shift a production frontier. Fourth, the production of population health is a multifactorial and complex issue, so it is practically impossible to make the absolutely right choice of key health system inputs [33].

In the future research, we should model the health system as a heterogeneous system that is composed of several interconnected divisions. Since the number of countries is limited, the network models will also allow us to use more inputs and outputs than conventional DEA models. Therefore, we have to prioritise using of the network DEA models with more input and output indicators in the health system efficiency evaluation. Another promising way of the future research in the health system efficiency evaluation is to apply a conditional order-*m* DEA models [34] that are able to take into account external factors and are much more robust to outliers than full frontier estimators.

## Data Availability

The OECD dataset analysed during this study (Table 1) is publicly available from 10.1787/health-data-en. The HAQ Index is available from publication [26].
